# Capability of Human Dendritic Cells Pulsed with Autologous Induced Pluripotent Stem Cell Lysate to Induce Cytotoxic T Lymphocytes against HLA-A33-Matched Cancer Cells

**DOI:** 10.3390/ijms232112992

**Published:** 2022-10-27

**Authors:** Tsutomu Nakazawa, Ryosuke Maeoka, Takayuki Morimoto, Ryosuke Matsuda, Mitsutoshi Nakamura, Fumihiko Nishimura, Shuichi Yamada, Ichiro Nakagawa, Young-Soo Park, Hiroyuki Nakase, Takahiro Tsujimura

**Affiliations:** 1 Department of Research and Development, Grandsoul Research Institute for Immunology, Matsui 8-1, Utano, Uda 633-2221, Nara, Japan; 2 Clinic Grandsoul Nara, Matsui 8-1, Utano, Uda 633-2221, Nara, Japan; 3 Department of Neurosurgery, Nara Medical University, Kashihara 634-8522, Nara, Japan

**Keywords:** cancer vaccine, dendritic cell (DC), induced pluripotent stem cell (iPSC), cytotoxic lymphocyte (CTL), cancer immunotherapy

## Abstract

Irradiated murine induced-pluripotent stem cells (iPSCs) elicit the antitumor response in vivo. However, it is unclear whether human iPSCs would elicit antitumor effects. In the present study, we investigated the capability of human iPSC lysate (iPSL)-pulsed dendritic cells (DCs) (iPSL/DCs) to induce cancer-responsive cytotoxic T lymphocytes (CTLs) in vitro. iPSCs and DCs were induced from peripheral blood mononuclear cells isolated from a human leukocyte antigen (HLA)-A33 homozygous donor. The iPSL was pulsed with immature DCs, which were then stimulated to allow full maturation. The activated DCs were co-cultured with autologous CTLs and their responses to SW48 colorectal carcinoma cells (HLA-A32/A33), T47D breast cancer cells (HLA-A33/A33), and T98G glioblastoma cells (HLA-A02/A02) were tested with enzyme-linked immunospot (ELISPOT) assays. Comprehensive gene expression analysis revealed that the established iPSCs shared numerous tumor-associated antigens with the SW48 and T47D cells. Immunofluorescent analysis demonstrated that the fluorescent-labeled iPSL was captured by the immature DCs within 2 h. iPSL/DCs induced sufficient CTL numbers in 3 weeks for ELISPOT assays, which revealed that the induced CTLs responded to SW48 and T47D cells. Human iPSL/DCs induced cancer-responsive CTLs on HLA-A33-matched cancer cells in vitro and could be a promising universal cancer vaccine for treating and preventing cancer.

## 1. Introduction

Harnessing the immune system to eradicate malignant cells is becoming a powerful new approach to cancer therapy. Checkpoint receptor antibodies that include the programmed cell death (PD)-1 antibody for treating multiple cancers have greatly advanced basic research and clinical studies in cancer immunotherapy. Despite the success of checkpoint blockade therapy, more than 50% of patients with cancer nevertheless fail to respond to it [1].

The advent of new technologies such as next-generation sequencing has enhanced our ability to search for new immune targets in onco-immunology and has accelerated immunotherapy development with a potentially broader coverage of patients with cancer. Recently, several immunotherapy approaches targeted the antigens encoded by tumor-specific mutated genes (neoantigens) derived via whole-genome sequencing from mutations unique to individual patients’ tumors [2]. Neoantigens are newly synthesized in tumors and recognized as non-self. Immune-mediated tumor rejection is associated with cytotoxic responses to neoantigen-derived peptides in a noncovalent association with self-human leukocyte antigen (HLA) molecules. By targeting neoantigens, T cells can attack and kill cancer cells [3,4,5]. There is strong evidence that neoantigen-based therapies, such as adoptive T-cell therapy, have the potential to induce remission in patients with treatment-resistant metastatic breast cancer [6].

Neoantigen-based cancer vaccines are similarly designed to elicit or amplify antigen-specific T-cell populations and stimulate directed antitumor immunity, but neoantigen selection and prioritization remain challenging. Bioinformatic algorithms can predict tumor neoantigens from somatic mutations, insertion–deletions, and other aberrant peptide products, but this often leads to hundreds of potential neoepitopes unique to the tumor [2]. Designing hundreds of neoantigen-related peptides in accordance with a patient with cancer is difficult in terms of cost and time. Furthermore, the tumor-associated antigens (TAAs) including neoantigens that will be expressed in cancer tissues in the future cannot be targeted.

With their large amounts of characterized and uncharacterized T-cell epitopes available for activating CD4+ T helper (Th) and CD8+ cytotoxic lymphocytes (CTLs) simultaneously, whole-tumor antigens in tumor cells represent an attractive alternative source of antigens to full-length recombinant tumor proteins and tumor-derived peptides [7,8]. Dendritic cell (DC) vaccines are a promising method for generating a therapeutic antitumor immune response. DCs are professional antigen-presenting cells and can initiate T cells function. DCs derived in vitro from peripheral blood mononuclear cells (PBMCs) can be primed against tumor antigens in culture; upon subsequent vaccination in tumor-bearing hosts, they can elicit antitumor immunity [9]. DCs loaded with patient-derived cancer cell lysates are a feasible treatment in several cancers [10]; however, these are limited by tumor burden. If abundant TAA-expressing cells were established, they could be an ideal material for DC vaccines.

Embryonic stem cells (ESCs) and induced pluripotent stem cells (iPSCs) are remarkably similar to cancer cells. Cancer cells share many cellular and molecular features with ESCs [11], including a rapid proliferation rate [12], upregulated telomerase activity [13], increased expression levels of oncogenes such as c-*MYC* [14] and Krüppel-like factor 4 (*KLF4*) [15], similar overall gene expression profiles [16,17], microRNA signatures [18], and epigenetic statuses [19]. These ESC features resemble the hallmarks of cancer cells that have sustained proliferative and immortality abilities [20]. The discovery of iPSCs [21,22] revolutionized stem cell research and the scientific field. Reprogrammed human iPSCs from somatic tissues share near identical gene expression profiles as ESCs [23,24,25,26], which presents a possible solution to the ethical objections against the use of human ESCs in many countries. Similar to ESCs, iPSCs share genetic and transcriptomic characteristics with cancer cells [27]. Human iPSCs were first generated by the transduction of fibroblasts with four transcription factors: *OCT4*, *SOX2*, c-*MYC*, and *KLF4* [22]. c-*MYC* is a well-known oncogene [26,28] and the other three factors are upregulated in multiple cancer types [29,30,31,32,33,34]. Moreover, these genes are associated with tumor progression and poor prognosis in certain tumor types [35], which suggests that targeting these genes in cancers could be therapeutically beneficial. Recently, it was reported that irradiated iPSCs elicited an anti-tumor response in murine melanoma via the immune system in vivo as the iPSCs expressed large amounts of TAAs with several cancer cells [27,36]. However, it remains unclear whether human iPSCs elicit the antitumor response via the induction of cancer-responsive T cells.

The administration of irradiated human iPSCs to humans in vivo is ethically controversial and difficult to perform immediately. Therefore, as a first step, we investigated whether iPSCs could induce cancer-cell-responsive T cells through DCs in vitro. This approach would also confirm the feasibility of the clinical application of iPSC-pulsed DC vaccination for cancer immunotherapy.

## 2. Results

### 2.1. Human iPSCs Expressed Tumor-Specific and Tumor-Associated Antigens

**Figure 1:**
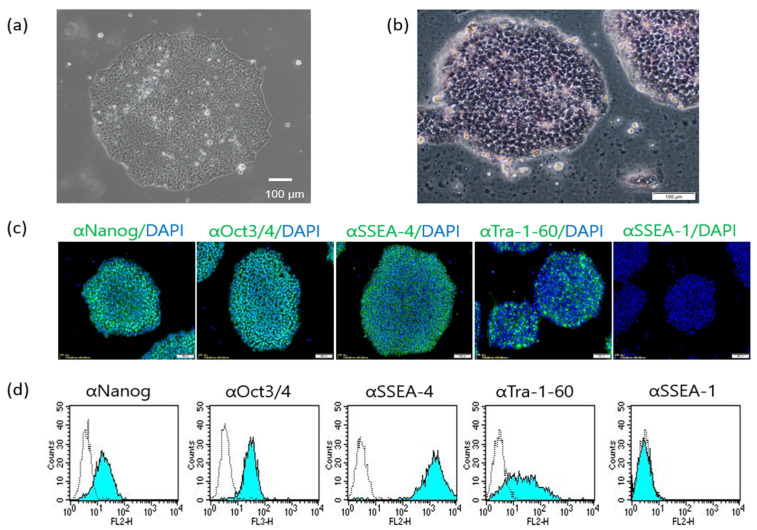
Stem cell marker expression in the established colonies. (**a**) The colonies underwent > 10 passages. (**b**) Alkaline phosphatase staining. Pink indicates positive alkaline phosphatase staining. (**c**) Immunostaining of stem cell markers expressed in and on the colonies. Green (green fluorescence) and blue (DAPI) indicate positive staining for individual antigens and the nucleus, respectively. (**d**) Flow cytometric analysis of stem cell markers. Light blue and white histograms depict specific antigens and isotype controls, respectively. White bar = 100 µm in all photos.

We attempted to establish iPSCs derived from human peripheral blood using episomal gene expression vectors carrying six genes *(OCT3*/*4*, *KLF4*, *SOX2*, L-*MYC*, *LIN28,* and mouse p53 dominant negative form). Approximately 20 days later, we observed distinct colonies that were flat and resembled human iPSC colonies. The colonies were picked up, disaggregated into single cells with enzymatic digestion, and passaged > 10 times, and they exhibited a similar morphology to human iPSCs (Figure 1a) and expressed alkaline phosphatase (Figure 1b). The pluripotent stem cell marker expression analysis revealed that the colonies expressed human-ESC-specific surface antigens, including SSEA-3, SSEA-4, and TRA-1-60 and the nuclear proteins NANOG and OCT3/4. The colonies did not express SSEA-1 (Figure 1c,d). Generally, except for a few cells at the edge of the colonies, the human iPSCs did not express SSEA-1 [22,37].

**Figure 2:**
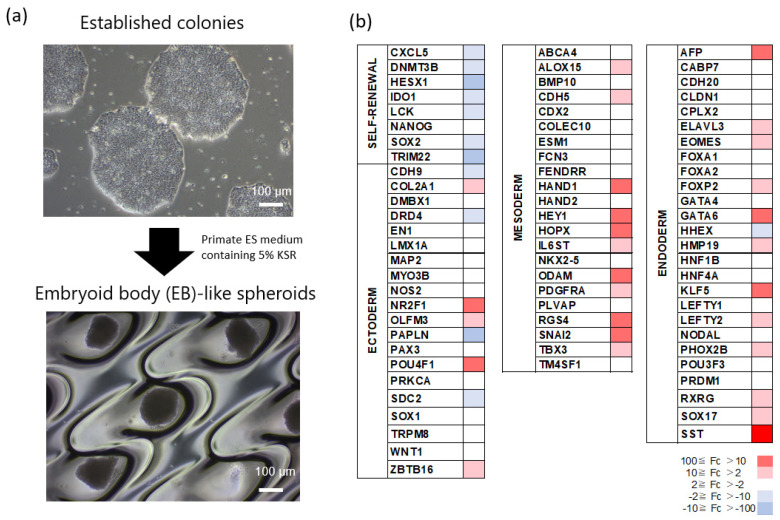
Relative expression of self-renewal and differentiation genes in EB-like spheroids derived from the established colonies. (**a**) EBs induced by the established colonies in the specific culture conditions. White bar = 100 µm. (**b**) Change in gene expression during EB-like spheroid formation with the established colonies. Gene expression of EB-like spheroids formed from the established colonies in 7-day cultures was determined using microarray analysis. Gene expression was reported as the relative fold change between the established colonies and EB-like spheroids. The change from red to light purple indicates fold changes (Fc) from 100 to 10, 10 to 2, 2 to −2, −2 to −10, and −10 to −100.

To determine the differentiation ability of human iPSCs in vitro, we generated EBs using suspension cultures as described previously with brief modifications [21,38]. After cultivation in suspension culture, the colony cells formed sphere structures that were similar to EBs (Figure 2a). Microarray analysis of the gene expression of the EB-like spheroids as compared to the established colonies demonstrated that the EB-like spheroids had downregulated self-renewal-related genes (*CXCL5*, *DNMT3B*, *HESX1*, *IDO1*, *LCK*, *SOX2*, and *TRIM22,* >2-fold changes), and upregulated ectoderm-associated genes (*COL2A1*, *NR2F1*, and *ZBTB16,* >2-fold changes), mesoderm-related genes (*ALOX15*, *CDH5*, *HAND1*, *HEY1*, *HOPX*, *IL6ST*, *ODAM*, *PDGFRA*, *RGS4*, *SNAI2*, and *TBX3,* >2-fold changes), and endoderm-associated genes (*AFP*, *ELAVL3*, *EOMES*, *FOXP2*, *GATA6*, *HMP19*, *KLF5*, *LEFTY2*, *PHOX2B*, *RXRG*, *SOX17*, and *SST,* >2-fold changes) (Figure 2b). These results demonstrated that the established colonies represented pluripotency and could differentiate into the three germ layers and were defined as iPSCs.

### 2.2. The Established iPSCs-Expressed TAAs

**Figure 3:**
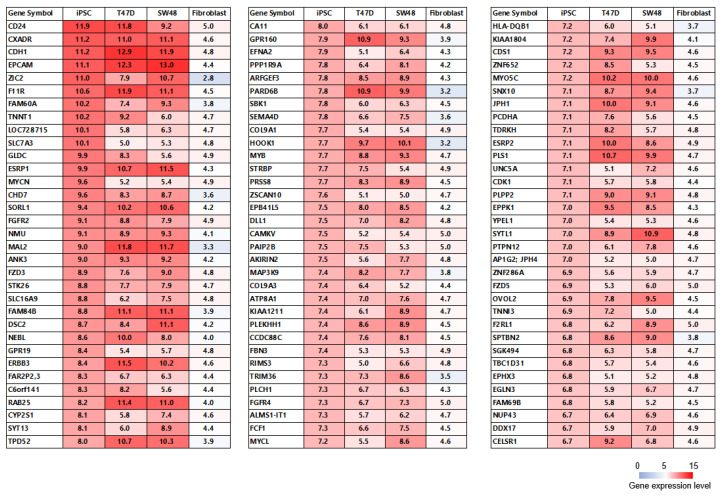
Heatmap analysis of gene expression of iPSCs, SW48 and T47D cells, and fibroblasts. The iPSC data were obtained from microarray analysis. The SW48 and T47D cell and fibroblast data were obtained from the NCBI GEO database. The listed genes are highly expressed in iPSCs and are very lowly or not expressed in fibroblasts (gene expression level < 5). The change from red to light purple indicates the gene expression level changing from 15 to 0. The 99 genes in the list were selected in order of highest gene expression in the iPSCs.

**Figure 4:**
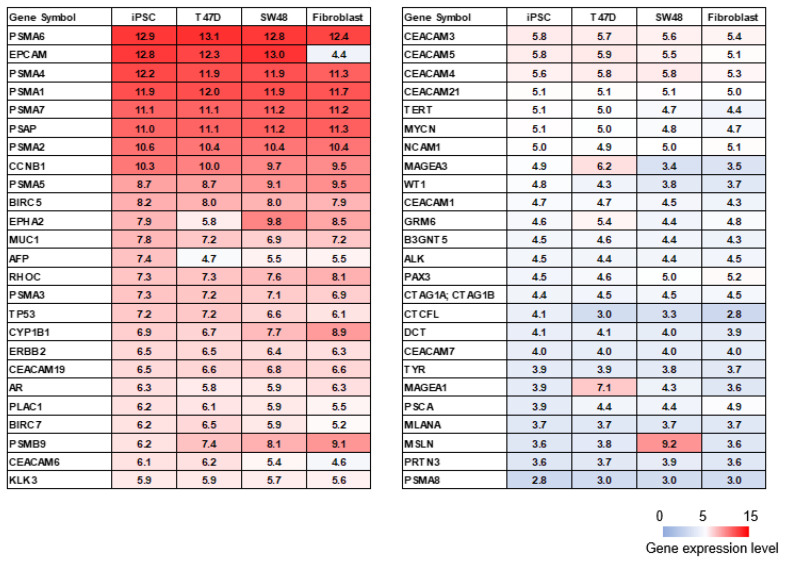
TAAs selected by the NCI Pilot Project. The list shows the ranking of TAAs selected as described previously [39]. The iPSC data were obtained using microarray analysis. The iPSC, SW48 and T47D cell, and fibroblast data were obtained from the NCBI GEO database. The change from red to light purple indicates the gene expression level changing from 15 to 0.

We performed microarray analysis on the established human iPSCs to compare the expression profiles of T47D (human breast cancer) and SW48 cells (human colorectal cancer) and fibroblasts from the National Center for Biotechnology Information (NCBI) GEO database. The high-expression genes (gene expression levels > 5) in the iPSCs and T47D and SW48 cells and low-expression genes in the fibroblasts (gene expression levels ≤ 5) were selected. The 99 genes that were commonly highly expressed in iPSCs and T47D and SW48 cells but with low expression in fibroblasts are depicted in Figure 3. The heat map depicts the genes included in the cancer-related antigen ranking reported by the US National Cancer Institute (NCI), which were selected based on therapeutic function, immunogenicity, antigen role in oncogenicity, specificity, expression level and percent of antigen-positive cells, stem cell expression, number of patients with antigen-positive cancers, number of antigenic epitopes, and cellular location of antigen expression [39]. The iPSCs also expressed *PSMA1*, *PSMA2*, *PSMA4*, *PSMA6*, *PSMA7*, *EPCAM*, *PSAP*, and *CCNB1*, which were present in the NCBI cancer antigen ranking (Figure 4).

### 2.3. Autologous iPSL/DCs-Induced Tumor-Cell-responsive CTLs Derived from a HLA-A33 Donor

**Figure 5:**
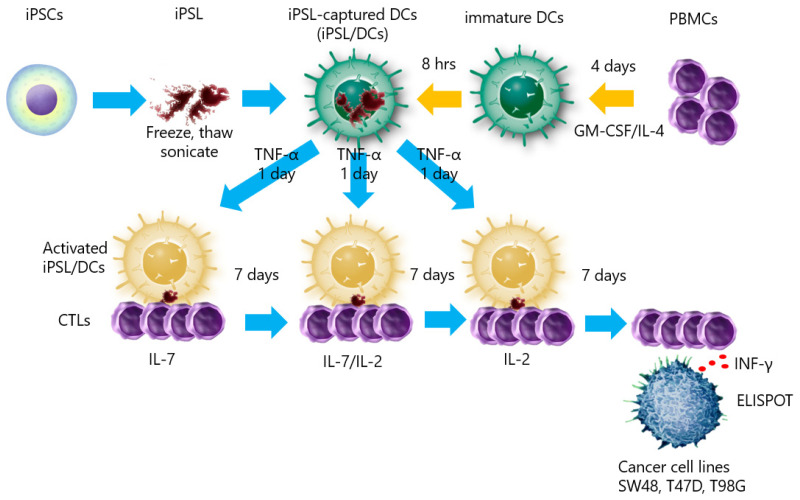
Schematic representation of CTLs induced by iPSL/DCs. iPSL was prepared by freeze–thawing and sonication. DCs were induced by rhGM-CSF and rhIL-4 for 4 days. The immature DCs were loaded with iPSL for 8 h and activated for 24 h with rhTNF-α. The iPSL/DCs were co-cultured with CTLs in the presence of rhIL-7, re-stimulated by iPSL/DCs, rhIL-2, or rhIL-7 weekly, and cultured for 3 weeks.

To determine whether human iPSCs could induce tumor-responsive CTLs, we prepared iPSL/DCs generated from peripheral blood cells carrying HLA-A33 and co-cultured them with CD8+ T lymphocytes in vitro. The CD8+ T lymphocytes were reacted with cancer cell lines with or without HLA-A33 (Figure 5).

**Figure 6:**
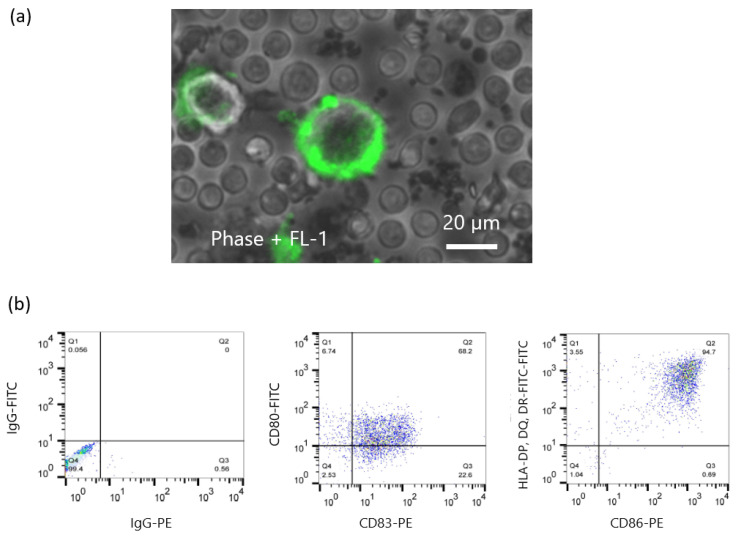
Immature DCs-captured iPSL and phenotyping characterization of activated iPSL/DCs. (**a**) The photo depicts entrapped FITC-labeled annexin-V-binding iPSL in an immature DC. The photo was captured within 2 h after co-culture. (**b**) Flow cytometric characterization of TNF-α-activated DCs. The activated DCs expressed the activated markers CD80, CD83, CD86, and HLA-DR.

**Figure 7:**
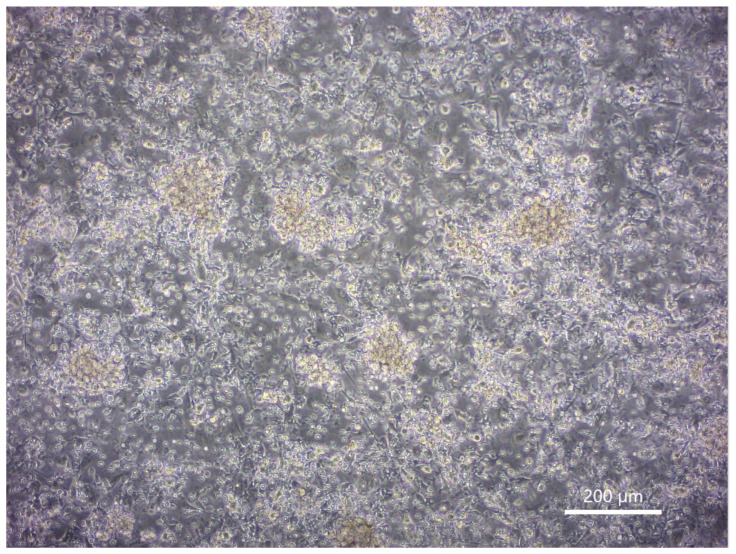
The CTLs induced by iPSL/DCs. The photo was taken from a 2-week co-culture of CTLs with iPSL/DCs. White bar = 200 µm.

**Figure 8:**
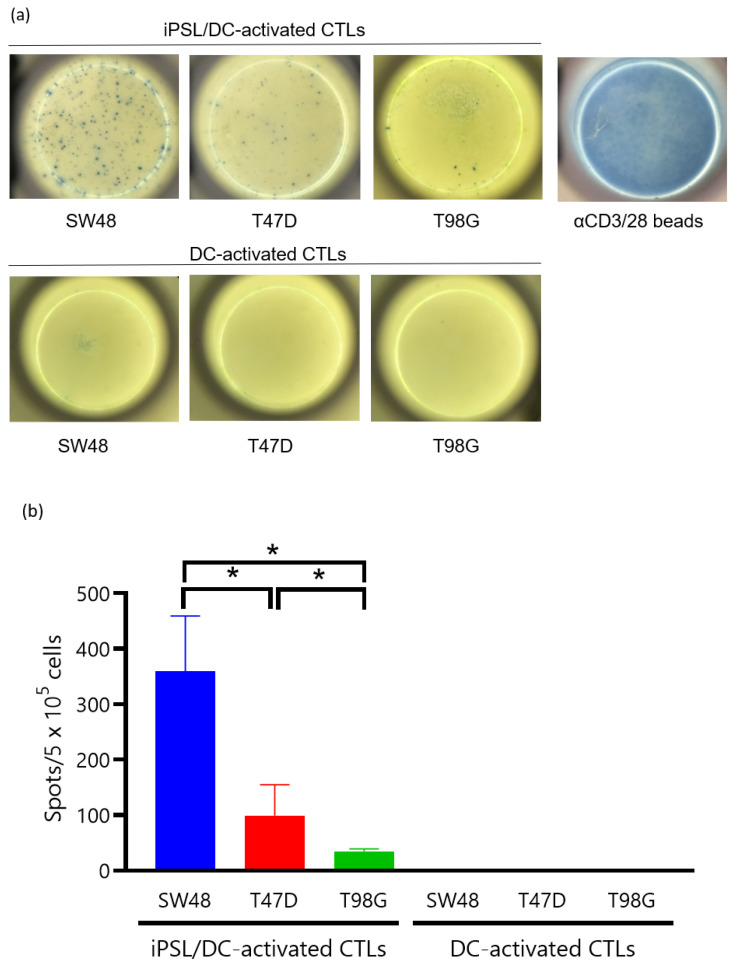
CTLs induced by iPSL/DCs respond to cancer cells carrying HLA-A33. (**a**) Representative photos of IFNγ-based ELISPOT assays. Top row depicts IFNγ-producing spots of iPSL/DC-induced CTLs co-cultured with cancer cells. Rightmost photo depicts the positive control of IFNγ-producing cells stimulated by anti-CD3 and anti-CD28 antibody. Bottom row depicts DC-induced CTLs. (**b**) The graph depicts the frequency of IFNγ-producing cells in 5 × 10^5^ CTLs. Data are the mean ± SD of 3–4 experiments with at least > 2 independent experiments. The significance of differences was determined by the Mann–Whitney U test. * *p* < 0.05.

iPSL was prepared by freeze–thawing and sonication. Before the CTL induction experiments, FITC-labeled annexin V-stained iPSL was co-cultured with immature DCs and recorded under phase-contrast, fluorescent inverted microscopy to confirm whether the DCs had captured the iPSL. Annexin V binds to phosphatidylserine in the cell membrane [40]. Accordingly, the iPSL bound by the FITC-labeled annexin V was captured by the immature DCs within 2 h (Figure 6a and Supplement Video S1). Subsequently, flow cytometry of the TNF-α-activated iPSL/DCs revealed that the cells were positive for CD80, CD83, CD86, and HLA class II expression (Figure 6b). The iPSL/DCs were co-cultured with CTLs, where the CTLs gathered around the DCs and activated (Figure 7). Following a 3-week culture, sufficient numbers of CTLs were obtained for ELISPOT assays, which revealed that there were IFNγ-producing cells in 360 ± 87, 100 ± 48, and 35 ± 4 iPSL/DC-activated CTLs per 5 × 10^5^ CTLs co-cultured with SW48, T47D, and T98G cells, respectively. SW48 and T47D-responding CTLs were increased compared to T98G-responding CTLs. By contrast, the IFNγ-producing cells in the DC-activated CTLs without iPSL pulsing were not detectable against each cell line. These results indicated that iPSL/DCs induced CTLs that reacted with cancer cells carrying HLA-A33 as compared to CTLs that reacted with cell lines without HLA-A33 (Figure 8).

## 3. Discussion

The concept of irradiated murine iPSCs as a prophylactic cancer vaccine has been evaluated and elicited antitumor effects in lung cancer [41,42], melanoma [27,43], and pancreatic ductal adenocarcinoma [36]. These studies revealed that ESCs also present immunogenic features [44,45], where the results implied that the vaccines should be evaluated in humans. However, vaccinating humans with irradiated human iPSCs and ESCs is ethically controversial and challenging to perform immediately. Therefore, we investigated as an initial step whether human iPSCs can induce a CTL response to cancer cells via DCs in vitro. We considered that the experiment could at least present the possibility that human iPSCs contain abundant functional TAAs that would generate cancer immune responses. To our knowledge, this is the first report of iPSL/DCs, where both cells were derived from human PBMCs carrying HLA-A33, and the induced autologous CTLs responded to HLA-A33-carrying human cancer cells. Our experiment demonstrated that the iPSL/DCs induced cancer-responsive CTLs, which strongly responded to SW48 cells and responded less strongly to T47D cells. The CTL response to T98G cells was extremely low. This result indicated that the iPSL/DCs elicited CTLs that responded to HLA-A33-matched cancer cells. DCs without iPSL pulsing did not induce CTLs against any of the tested cancer cells. These results implied that the iPSL/DCs induced cancer-responsive CTLs in an HLA-A33-unrestricted manner, albeit slightly.

DC vaccines are a promising method of generating a therapeutic antitumor immune response. DCs are professional antigen-presenting cells and can initiate CTL function. DCs derived in vitro from PBMCs can be primed against tumor antigens in culture and can elicit antitumor immunity upon subsequent vaccination in tumor-bearing hosts [46]. DC therapy is a safe and well-tolerated immunotherapeutic method that can elicit immunity even in patients with advanced-stage cancer [10] and can obtain a clinical response, yet not in all patients with cancer, such as melanoma, prostate cancer, malignant glioma, and renal cell carcinoma [46,47].

Although generating autologous iPSCs for each patient appears to be less feasible and a prophylactic cancer vaccine currently appears to be less relevant to clinical medicine, the iPSL/DC-based cancer vaccine described in our study has significant merits as a future immune therapy in clinical settings under certain scenarios. In a prophylactic setting, the iPSC/DC vaccine can be generated to treat people at high risk for developing cancer, such as patients with hereditary chronic pancreatitis, Lynch syndrome, and Li–Fraumeni syndrome [48,49,50,51]. Such patients have a much higher likelihood of developing cancer in their lifetime and are potentially suitable candidates for prophylactic iPSL/DC cancer vaccines. Furthermore, the iPSL/DCs can be used as an adjuvant immunotherapy. As an adjuvant, the irradiated iPSC vaccine inhibited melanoma recurrence at the resection site and reduced metastatic tumor load [27]. The iPSL/DCs could be developed at diagnosis and made available in surgical or chemo/radiotherapy treatment of cancer. Under these situations, the clinical development of cancer vaccines using the iPSL/DCs described in our study is warranted.

Human iPSCs share abundant TAAs in several cancer cells [27,36]. Here, we demonstrated that genes that were commonly highly expressed in iPSCs and T47D and SW48 cells were lowly expressed in fibroblasts (Figure 3). *CD24*, *CXADR*, *CDH1*, *EPCAM*, *ZIC2*, *F11R*, *FAM60A*, *TNNT1*, *LOC728715*, SLC7A3, *GLDC*, *ESRP1*, *MYCN*, *CHD7*, *SORL1*, *FGFR2*, *NMU*, *MAL2*, *ANK3*, and *FZD3* were the top 20 high-expression genes in the iPSCs and were candidates for TAAs in iPSL/DC vaccination. The iPSCs also expressed *PSMA1*, *PSMA2*, *PSMA4*, *PSMA6*, *PSMA7*, *EPCAM*, *PSAP*, and *CCNB1*, which were in the NCBI cancer antigen rankings (Figure 4). These results indicated that the previously reported candidate TAAs were abundant in iPSCs. However, the expression of Wilms tumor 1 (*WT1*), the most useful cancer-related antigen in the NCBI ranking, is very low in iPSCs [39]. On the other hand, if iPSCs expressed the same genes as normal cells, then the iPSC-reactive CTLs could attack normal cells. This point is a crucial point of iPSCs or ESCs used for the TAA source. We do not believe the point to be a major issue. The central positive selection in the thymus occurs in vivo to prevent normal cells from being attacked by self-active T cells [52]. Therefore, it is highly possible that iPSCs cannot induce self-reactive T cells in vivo, which ensures safety.

Taken together, our data demonstrate the feasibility of obtaining broad tumor immunity against multiple cancers using an iPSL/DC vaccine that presents the immune system with large quantities of tumor antigens. Compared to current immunotherapy strategies, our iPSL/DC vaccine can be generated within a few weeks after diagnosis without surgery. Given these advantages, iPSC/DC vaccines could be an option for personalized immunotherapy immediately following primary treatment of conventional cancers. Moreover, iPSC/DC vaccines can be generated to prevent high-risk individuals from developing cancer.

Our study has some limitations. First, we only evaluated the autologous setting in a donor carrying HLA-A33. If iPSL/DCs are to be applied to a wider range of patients, it is necessary to evaluate the reactivity of donors with other HLA phenotypes and not only HLA-A33. In addition, the CD8+ CTL response was limited; therefore, confirming CD4+ Th cells is an important issue to be addressed in the future. Further, more extensive in vivo confirmation using humanized mice should be needed. Second, we only evaluated autologous iPSCs. Allogenic iPSCs are a more attractive tool in iPSL/DC vaccination as iPSC libraries are stocked and available in several countries. However, autologous iPSCs may provide a more accurate and representative panel of a patient’s tumor immunogens than allogenic iPSCs. We believe that resolving these issues would present the possibility of conducting future clinical trials.

## 4. Conclusions

Human iPSL/DCs induced cancer-responsive CTLs in an HLA-A33 dependent manner in vitro. iPSL/DCs could be a promising universal cancer vaccine against several cancers and a prophylactic cancer vaccine.

## 5. Materials and Methods

### 5.1. Ethics

Peripheral blood was collected from a healthy volunteer with the approval of the Nara Medical University Ethics Committee (No. 1058) and in accordance with its guidelines. Informed consent was obtained according to the tenets of the Declaration of Helsinki.

### 5.2. Peripheral Blood Mononuclear Cells

The PBMCs were prepared by density gradient centrifugation (Lymphoprep; Axis-Shield PoC AS, Oslo, Norway). HLA typing was performed using PCR-sequencing-based typing (PCR-SBT) at Special Reference Laboratory (Tokyo, Japan).

### 5.3. Human Cell Lines

The SW48 (colorectal cancer: HLA-A33), T47D (breast cancer: HLA-A33), and T98G (glioblastoma: HLA-A02) cell lines were obtained from American Type Culture Collection (Manassas, VA, USA). The cells were maintained in Dulbecco’s modified Eagle’s medium (DMEM; Thermo Fisher Scientific, Waltham, MA, USA) supplemented with 10% heat-inactivated fetal bovine serum (FBS; MP Biomedicals, Tokyo, Japan), 100 U/mL of penicillin, and 100 µg/mL of streptomycin (Thermo Fisher Scientific) at 37 °C in a humidified atmosphere containing 5% CO_2_.

### 5.4. Generation of iPSCs and Lysate Production

#### 5.4.1. iPSC Generation and Passage

iPSCs were established from PBMCs from an HLA-A33 homozygous donor using Human iPS Cell Generation Episomal Vector Mix (Takara Bio, Shiga, Japan) with reference to the protocol of the Center for iPS Cell Research and Application (CiRA) of Kyoto University, Japan.

Briefly, episomal vectors (OCT3/4, KLF4, SOX2, L-MYC, and LIN28 mouse p53 dominant negative form) were electroporated to 2 × 10^5^ PBMCs using a Human T Cell Nucleofector Kit (Lonza, Basel, Switzerland). The electroporation was performed by Nucleofector 2B (Lonza), program number V-024. The electroporated cells were cultured in AIM-V medium (Thermo Fisher Scientific) supplemented with 200 IU/mL of recombinant human interleukin-2 (rhIL-2; Primmune Inc., Kobe, Japan) in 6-well plates coated with human laminin 511E8 fragment (iMatrix-511; Nippi, Tokyo, Japan). Then, Human T-Activator CD3/CD28 (Thermo Fisher Scientific) was added for T cell activation. The medium was replenished with StemFit AK02N (Ajinomoto, Tokyo, Japan) on days 2, 4, and 6, and the culture medium was completely changed to StemFit AK02N on day 8. Thereafter, the medium was changed every 2 days.

Colonies appeared after 20 days and were picked up by pipettes under a stereomicroscope (SZ61, Olympus, Tokyo, Japan). The colony-derived cells were incubated for 10 min in TrypLE select (Thermo Fisher Scientific) to prepare for single-cell suspensions. The cells were cultured in StemFit AK02N supplemented with 10 µM ROCK inhibitor (Y-27632; FUJIFILM Wako, Tokyo, Japan) in 6-well plates coated with human laminin 511E8 fragment. The next day, the medium was changed to fresh StemFit AK02N without ROCK inhibitor. The medium was changed to every 2 days until the cells were confluent. The cellular characterization was performed after >10 passages.

#### 5.4.2. Cellular Characterization of iPSCs

Alkaline phosphatase staining was performed on the culture plates with a Leukocyte Alkaline Phosphatase Kit based on naphthol AS-MX phosphate and fast blue RR salt (Sigma-Aldrich, MO, USA).

The immunofluorescent detection of the stem/progenitor cell markers was performed by flow cytometry and immunocytochemistry. In flow cytometry, the cells were stained with the following primary antibodies: phycoerythrin (PE)-conjugated mouse anti-human stage-specific embryonic antigen (SSEA)-4 (clone MC-480; BioLegend, San Diego, CA, USA), PE-conjugated mouse anti-human tumor-related antigen (TRA)-1-60-R (BioLegend), and PE-conjugated mouse anti-human SSEA-1 (MC-480, BioLegend). Intracellular NANOG and OCT3/4 expression was detected with a BD Pluripotent Stem Cell Transcription Factor Analysis kit (BD Biosciences, Franklin Lakes, NJ, USA). The flow cytometric data were acquired by a BD FACSCalibur unit (BD Biosciences) and analyzed using CellQuest software version 6.0 (BD Biosciences) or FlowJo (BD Biosciences).

In the immunocytochemistry, the primary antibodies were mouse anti-human SSEA-4 (813-70; Santa Cruz Biotechnology, TX, USA), mouse anti-human TRA-1-60 (MAB4360; Merck Millipore, Billerica, MA, USA), mouse anti-human SSEA-1 (MC480; BioLegend), rabbit anti-human NANOG (ReproCELL, Kanagawa, Japan), and mouse anti-human OCT3/4 (C-10; Santa Cruz Biotechnology). The secondary antibodies were Alexa Fluor 488-conjugated goat anti-mouse IgG (H+L) (Thermo Fisher Scientific) and Alexa Fluor 488-conjugated goat anti-rabbit IgG H&L (Abcam, Cambridge, UK). Nuclear staining was performed using 4′,6-diamidino-2-phenylindole dihydrochloride (DAPI; Dojin, Kumamoto, Japan). Phase-contrast and fluorescent images were captured by an IX83 inverted microscope (Olympus) and analyzed with cellSens imaging software (Olympus).

#### 5.4.3. Preparation of iPS Lysate

iPSC lysate (iPSL) was prepared as previously reported [47]. Briefly, iPSCs were harvested from confluent culture flasks and resuspended at 1 × 10^7^/mL in phosphate-buffered saline (PBS). The iPSL was produced via five freeze–thaw cycles and subsequent sonication by a Q700 sonicator (Qsonica, Newtown, CT, USA) to produce a homogeneous lysate. The sonication setting was as follows: amplitude 20 and pulse time 7 s. For confirmation of iPSL phagocytosis by DCs, 2 × 10^5^ iPSLs was stained with 20 µL of fluorescein isothiocyanate (FITC)-labeled annexin V (BioLegend).

### 5.5. Comprehensive Gene Expression Analysis

#### 5.5.1. RNA Extraction and Microarray Gene Expression Assay

The total RNA of the cells was extracted with a NucleoSpin RNA kit (MACHEREY-NAGEL, Düren, Germany). The gene expression of the RNA samples was analyzed by Takara Bio using the Affymetrix Human Genome U133 Plus 2.0 Array (Affymetrix, Santa Clara, CA, USA). The microarray data were obtained from the established iPSCs and embryoid bodies (EBs) and deposited in the Gene Expression Omnibus (GEO) database. The iPSC and EB accession numbers were GSE213687, respectively.

#### 5.5.2. Differentiation Ability of iPSCs into Ebs

Ebs were induced from iPSCs in Primate ES Cell Medium (ReproCELL) supplemented with KnockOut™ Serum Replacement (Thermo Fisher Scientific) at an EZSPHERE 6-well plate (IWAKI, Sizuoka, Japan) for 7 days. Differentiation ability was confirmed by the expression of three germ layer markers in the Ebs via microarray gene expression assays. Differential expression analysis was performed by Transcriptome Analysis Console (TAC) software (Thermo Fisher Scientific) using the gene set below. These gene contents were based on published work and were validated against multiple human ES and iPS lines [23].

Self-renewal: CXCL5, DNMT3B, HESX1, IDO1, LCK, NANOG, SOX2, TRIM22.

Ectoderm: CDH9, COL2A1, DMBX1, DRD4, EN1, LMX1A, MAP2, MYO3B, NOS2, NR2F1, OLFM3, PAPLN, PAX3, POU4F1, PRKCA, SDC2, SOX1, TRPM8, WNT1, ZBTB16.

Mesoderm: ABCA4, ALOX15, BMP10, CDH5, CDX2, COLEC10, ESM1, FCN3, FENDRR, HAND1, HAND2, HEY1, HOPX, IL6ST, NKX2-5, ODAM, PDGFRA, PLVAP, RGS4, SNAI2, TBX3, TM4SF1.

Endoderm: AFP, CABP7, CDH20, CLDN1, CPLX2, ELAVL3, EOMES, FOXA1, FOXA2, FOXP2, GATA4, GATA6, HHEX, HMP19, HNF1B, HNF4A, KLF5, LEFTY1, LEFTY2, NODAL, PHOX2B, POU3F3, PRDM1, RXRG, SOX17, SST.

#### 5.5.3. Comparison of the Expression Patterns of Tumor-associated Genes in iPSCs

The comprehensive gene expression data of the iPSCs were obtained from GEO database accession numbers GSM829481 and GSM829481, GSM274652 and GSM2318773 (T47D cells), GSM1400240 and GSM844713 (SW48 cells), and GSM1868517 and GSM1868518 (fibroblasts). Differential expression analysis was performed with TAC software.

### 5.6. Preparation of iPSL-Loaded DCs and CTL Induction

To prepare the DCs, 5 × 10^6^ PBMCs carrying HLA-A33 from a homozygous donor were suspended in AIM-V medium supplemented with 10% autologous plasma in a 6-well plate (Corning, CA, USA). The cells were enriched by adherence and cultured with AIM-V supplemented with 800 IU/mL of rh granulocyte–monocyte colony-stimulating factor (rhGM-CSF; Primmune Inc.) and 500 IU/mL of rhIL-4 (Primmune Inc.). After 2 days, 800 IU/mL of rhGM-CSF and 500 IU/mL of rhIL-4 were added and cultured for another 2 days.

Immature DCs (2 × 10^5^) were loaded with 6 × 10^5^ iPSLs in AIM-V medium containing 10% autoplasma for 8 h and activated by a 24 h culture with 20 ng/mL of rh tumor necrosis factor alpha (rhTNF-α; Miltenyi Biotech, Bergisch Gladbach, Germany). The activated DCs were stained with FITC-conjugated anti-human CD80 (clone B7-1, BD Biosciences), PE-conjugated anti-human CD83 (BL11, BD Biosciences), PI-conjugated anti-human CD86 (B70/B7-2, BD Biosciences), and FITC-conjugated anti-human HLA-DP, DQ, and DR (Tü39, BD Biosciences). Flow cytometric analysis was performed with a BS FACSMelody unit (BD Biosciences). Paired IgG isotype controls (BD Biosciences) were used.

The 8 × 10^4^ iPSL-pulsed DCs (iPSL/DCs) were co-cultured with 2 × 10^6^ purified CD8+ T cells isolated with an EasySep Human CD8+ T Cell Isolation Kit (STEMCELL Technologies, Vancouver, Canada) in the presence of 10 ng/mL of rhIL-7 (Miltenyi Biotech) and re-stimulated by 8 × 10^4^ iPSL/DCs, or 10 IU/mL of rhIL-2 or rhIL-7 weekly and cultured for 3 weeks.

### 5.7. ELISPOT Assays

Interferon (IFN) γ-secreting cells were detected with a Human IFNγ Single-Color Enzyme-Linked ImmunoSpot (ELISPOT) kit (Immunospot; Cellular Technology Limited, Shaker Heights, OH, USA) according to the manual. Briefly, nitrocellulose plates were coated with mouse anti-human IFNγ antibody overnight at 4 °C. The wells were washed with PBS and iPSL/DC-stimulated CTLs were co-cultured with SW48, T47D, or T98G cells at an effector-to-target cell ratio of 2:1. The positive control was IFNγ released by CTLs stimulated with CD3/CD28 beads (Thermo Fisher Scientific). After 20 h of incubation at 37 °C and 5% CO_2_, staining and color development were performed. Color spots were determined by counting under stereomicroscopy.

### 5.8. Statistics

Statistical analyses were performed using GraphPad Prism 8 (GraphPad Software Inc., La Jolla, CA, USA). The data are reported as the mean ± standard deviation (SD). The significance of differences was determined by the Mann–Whitney U test. We considered *p* < 0.05 statistically significant.

## Data Availability

The data supporting the findings of this study are available from the corresponding author upon reasonable request.

## Supplementary Materials

The following supporting information can be downloaded at: https://www.mdpi.com/article/10.3390/ijms232112992/s1, Video S1: Immature DC captured iPSL. The video shows an immature DC entrapping FITC-labeled annexin V-binding iPSL. The video was captured under fluorescent and inverted microscopy 4 h after co-culture.
